# Inhalationally Administered Semifluorinated Alkanes (SFAs) as Drug Carriers in an Experimental Model of Acute Respiratory Distress Syndrome

**DOI:** 10.3390/pharmaceutics13030431

**Published:** 2021-03-23

**Authors:** Matthias Otto, Jörg Krebs, Peter Welker, René Holm, Manfred Thiel, Luciano Gattinoni, Michael Quintel, Charalambos Tsagogiorgas

**Affiliations:** 1 Department of Anaesthesiology and Critical Care Medicine, University Hospital Mannheim, Faculty of Medicine, University of Heidelberg, Theodor-Kutzer Ufer 1-3, 68165 Mannheim, Germany; matthias.otto@umm.de (M.O.); joerg.krebs@umm.de (J.K.); pwelker@ww-zahnaerzte.de (P.W.); manfred.thiel@medma.uni-heidelberg.de (M.T.); 2 Drug Product Development, Janssen Research and Development, Johnson & Johnson, Turnhoutseweg 30, 2340 Beerse, Belgium; rholm@ITS.JNJ.com; 3 Department of Science and Environment, Roskilde University, Universitetsvej 1, DK-4000 Roskilde, Denmark; 4 Department of Anesthesiology, Emergency and Intensive Care Medicine, University of Göttingen, Robert-Koch-Str. 40, 37075 Göttingen, Germany; gattinoniluciano@gmail.com (L.G.); mquintel@gwdg.de (M.Q.); 5 Department of Anesthesiology Donau-Isar-Klinikum, 94469 Deggendorf, Germany; 6 Department of Anaesthesiology and Critical Care Medicine, St. Elisabethen Hospital Frankfurt, Ginnheimer Straße 3, 60487 Frankfurt am Main, Germany

**Keywords:** fluorocarbons, aerosols, drug carriers, inhalation, respiratory distress syndrome, tissue distribution

## Abstract

Aerosol therapy in patients suffering from acute respiratory distress syndrome (ARDS) has so far failed in improving patients’ outcomes. This might be because dependent lung areas cannot be reached by conventional aerosols. Due to their physicochemical properties, semifluorinated alkanes (SFAs) could address this problem. After induction of ARDS, 26 pigs were randomized into three groups: (1) control (Sham), (2) perfluorohexyloctane (F6H8), and (3) F6H8-ibuprofen. Using a nebulization catheter, (2) received 1 mL/kg F6H8 while (3) received 1 mL/kg F6H8 with 6 mg/mL ibuprofen. Ibuprofen plasma and lung tissue concentration, bronchoalveolar lavage (BAL) fluid concentration of TNF-α, IL-8, and IL-6, and lung mechanics were measured. The ibuprofen concentration was equally distributed to the dependent parts of the right lungs. Pharmacokinetic data demonstrated systemic absorption of ibuprofen proofing a transport across the alveolo-capillary membrane. A significantly lower TNF-α concentration was observed in (2) and (3) when compared to the control group (1). There were no significant differences in IL-8 and IL-6 concentrations and lung mechanics. F6H8 aerosol seemed to be a suitable carrier for pulmonary drug delivery to dependent ARDS lung regions without having negative effects on lung mechanics.

## 1. Introduction

More than 50 years after its first description by Ashbaugh et al., the acute respiratory distress syndrome (ARDS) is still a life-threatening syndrome with mortality rates ranging from 11 to 87% [1,2]. Despite many years of basic scientific and clinical research, therapy remains symptomatic, trying to buy time for the mechanically ventilated lung to heal without causing additional harm by ventilator-induced lung injury (VILI) [3,4,5]. It has so far not been possible to identify a treatment of the cause.

Therapeutic interventions like low-tidal-volume ventilation, the use of low driving pressures, and early prone positioning have been shown to decrease mortality [6,7,8]. In contrast, a recent review demonstrated that until now no pharmacologic treatment addressing the pathomechanisms of ARDS has shown any survival benefit [5]. Even the approach of “direct” drug delivery to the lungs in the form of inhaled aerosols, a way to provide high organ-selective drug concentration while minimizing systemic side effects, has failed to improve patients’ outcomes [9,10,11,12].

Effectiveness of inhalation therapy in mechanically ventilated patients depends heavily on factors like the nebulizer model used, its operation mode, and position in the circuit [13]. More importantly, the pathophysiology of ARDS considerably limits successful drug delivery via inhalation therapy. Diffuse damage of the alveolo-capillary membrane, pulmonary hypertension, and high-protein pulmonary edema leads to increased lung tissue density causing collapse mainly in the most dependent lung regions [14,15,16]. Even after recruitment maneuvers, a reasonable part of the lungs remains poorly or nonaerated because of persistent atelectasis or even consolidation [17]. Nonaerated lung areas and alveoli filled with edema cannot be reached by a conventional aerosol; consequently, nebulized drugs are unlikely to be of therapeutic effect.

Semifluorinated alkanes (SFAs) used as excipients to transport aerosolized drugs to lung areas which cannot be reached by conventional aerosol application might offer a way to address this problem. SFAs are amphiphilic compounds consisting of fluorocarbon and hydrocarbon segments [18,19,20]. SFAs are chemically closely related to perfluorocarbons (PFCs) [21]. SFAs show low densities, low surface tension, and a positive spreading coefficient and are considered to be thermally, chemically, and biologically inert [18,20,21,22]. When used for liquid ventilation, PFCs as well as SFAs have been shown to reduce alveolar surface tension, remove alveolar exudates from dependent lung regions, and enable gas exchange [23,24]. An aerosol with these features might substantially increase the likelihood of an effective transport and deposition of drugs to dependent regions of ARDS lungs. Two in vivo studies investigating SFAs as a new class of excipients suitable for pulmonary drug delivery have already demonstrated the potential of perfluorohexyloctane (F6H8) and perfluorobutylpentane (F4H5) aerosols as lipophilic drug carriers [25,26].

The aim of the present study was to evaluate, in an experimental animal model of ARDS, whether an aerosol consisting of SFAs has the potential to transport and deposit a lipophilic drug like ibuprofen to dependent and nondependent lung regions. The effects of the aerosolized SFA F6H8 and F6H8 plus ibuprofen on inflammation were investigated by measuring the concentration of the proinflammatory cytokines TNF-α, IL-8, and IL-6 in the lung fluid. Lung mechanics were carefully monitored throughout the experiment.

## 2. Materials and Methods

### 2.1. Ethical Approval

This study was approved by the Institutional Review Board for the care of animal subjects (Regierungspräsidium Brandenburg, Frankfurt (Oder), Germany; Ref.-No: 23-2347-8-6-2008). All animals received humane care in compliance with the “Principles of Laboratory Animal Care” drawn up by the National Society for Medical Research, the “Guide for the Care and Use of Laboratory Animals” prepared by the National Academy of Sciences, USA, as well as the EC Directive 2010/63/EU, and the German Law for protection of vertebrate animals used for experimental and other scientific purposes.

### 2.2. Inhalation Formulations of Semifluorinated Alkanes

Medical grade inhalation SFA solutions with a purity grade of 99.8% based on perfluorohexyloctane (F6H8) with and without ibuprofen (F6H8 and F6H8-ibuprofen) were obtained from Novaliq GmbH (Heidelberg, Germany) ready for inhalation. Perfluorohexyloctane (F6H8) was used in the formulation, as it has previously been shown to be a suitable excipient for inhalation drug delivery [23,24]. The ibuprofen concentration in the formulation was 6 mg/mL. This was the highest possible ibuprofen concentration that could be dissolved in F6H8 without the use of a cosolvent.

All formulations were clear solutions without any precipitations.

### 2.3. Animal Study

The effects of nebulized F6H8 and F6H8-ibuprofen were investigated in a porcine ARDS model. Twenty-two female pigs (German landrace) were enrolled in this study and randomized into three different groups: control (sham, *n* = 7), F6H8 (*n* = 7), and F6H8-ibuprofen (*n* = 8). The average weight of the animals on the day of administration was 35.9 kg ± 5.0 kg, with no significant differences in weight between the three groups (*p* = 0.594).

#### 2.3.1. Anesthesia and Preparation of the Test Animals

Animals received an intramuscular (IM) premedication with 4 mg/kg azaperone and 0.01 mg/kg atropine 40 min, and 10 mg/kg ketamine 20 min prior to induction of general anesthesia, an IV line was placed into an ear vein. Anesthesia was induced with 5 mg/kg intravenous sodium thiopental and maintained with continuous infusion of midazolam (5–20 mg/kg/h) and fentanyl (1–10 µg/kg/h). The depth of anesthesia was carefully monitored through observing changes in heart rate or blood pressure and adjusted accordingly. Muscle relaxation was obtained with a bolus of 0.1 mg/kg pancuronium bromide followed by a continuous infusion of 0.1 mg/kg/h. A continuously applied balanced crystalloid solution with a flow rate of 0.1 mL/kg/min was used for fluid replacement. Fluid management was guided by measuring urine output via a urinary catheter inserted into the bladder.

Endotracheal intubation was performed with a 9.0 ID tube (Mallinckrodt Covidien, Dublin Ireland). The pigs were placed in supine position and ventilated by an Evita XL intensive care ventilator (Dräger Medical AG und Co. KGaA, Lübeck, Germany) in a volume control continuous mandatory ventilation mode (VC-CMV). Tidal volume (VT) was set to 6 mL/kg to ensure initial lung-protective ventilation. Respiratory rate was set at 20–30 breaths/min to maintain an arterial pCO_2_ in the range of 35–40 mmHg. I/E ratio was 1:1 and a positive end-expiratory pressure (PEEP) of 5 cm H_2_O with an inspiratory oxygen fraction (FiO_2_) of 1.0 was applied.

A PiCCO catheter (Getinge AB, Göteborg, Sweden) for the measurement of pulse contour cardiac output and for taking blood gas samples was placed into the femoral artery. For the measurement of intrathoracic blood volume (ITBV), extravascular lung water (EVLW), central venous pressure and pulmonal artery wedge pressure, and a central venous catheter (Arrow International Inc., Reading, PA, USA) as well as a pulmonary artery catheter were percutaneously inserted into the internal jugular vein. For assessing lung mechanics such as transpulmonary pressure, an esophageal catheter was placed according to the Baydur method [27,28].

#### 2.3.2. Induction of Acute Lung Injury

Acute lung injury was induced by repeated lavage-induced surfactant depletion with a body-warm saline solution until a PaO_2_/FiO_2_ ratio of < 100 mmHg was reached [29]. Pigs were than “injuriously” ventilated for one hour with a tidal volume of 10–12 mL/kg and zero end-expiratory pressure (ZEEP) to induce additional ventilator-induced lung injury (VILI).

### 2.4. Experimental Protocol

After induction of anesthesia and preparation of the animals, pre-ARDS baseline measurements of all test values, such as systemic and pulmonary hemodynamic parameters, ventilation parameters, lung mechanics, and blood gas samples were taken and indicated as “pre” in Table 1 and Table 2.

After successful induction of lung injury (defined as a PaO_2_/FiO_2_ < 100 mmHg) post-ARDS measurements were repeated and indicated as “post” in Table 1 and Table 2. Sixty min after the measurements, a recruitment maneuver was performed applying 40 cmH_2_O of pressure for 30 s. After recruitment, lung protective ventilation with a tidal volume of 6 mL/kg was resumed, and positive end-expiratory pressure (PEEP) was set to 10 cmH_2_O.

Aerosol therapy was performed using liquid inhalation of F6H8-solutions in a dosage of 1 mL/kg. While the control group (sham) received no inhalation, group two inhaled a pure F6H8 aerosol. Group three received F6H8-ibuprofen aerosol.

After baseline measurements, lung mechanic measurements were repeated at defined timepoints: 1, 5, 15, 30, 60, 120, 240, and 300 min after start of the aerosol administration. The last measurements after 300 min are indicated as “end” in Table 1 and Table 2. After the last measurements, all animals were euthanized in deep anesthesia using an overdose of sodium thiopental. Afterwards, bronchoalveolar fluid was collected via bronchoscopy (Ambu^®^ aScope™ slim, Bad Nauheim, Germany) from the right lung. Three 10 mL aliquots (30 mL in total) of sterile saline were instilled into the upper, middle, and lower lobe of the right lung. Aliquots were then retrieved by gentle suctioning through the suction port of the bronchoscope. The lavage samples were centrifuged for 10 min at 10,000 rpm (GPR, Beckman Instruments, Palo Alto, Santa Clara, CA, USA) to remove cells. The supernatant was then frozen using liquid nitrogen. The lungs were dissected en bloc. Lung tissue samples were taken from the very dorsal region of the upper, middle, and lower part of the right lung to verify and quantify the effects of the compounds applied.

### 2.5. Aerosol Generation and Delivery

F6H8- and F6H8-Ibuprofen solutions were aerosolized using an AeroProbe^®^ nebulization catheter (Trudell Medical International, London, ON, Canada) with a corresponding LABneb^®^ electromechanical catheter control system (Trudell Medical International, Ontario, Canada). The nebulization catheter consisted of a multilumen tubular shaft. The central lumen delivered the liquid formulation to be nebulized while the five peripheral lumens provided pressurized gas jets, which pneumatically aerosolized the liquid at the tip of the catheter. The catheter was operated with an input pressure of 60 psi (4.14 bar) on both the liquid lumen and the gas lumens with a total continuous flow of 1.4 L/min (manufacturer information), according to the settings already published in a preceding study [26].

### 2.6. Quantification of TNF-α, IL-6 and IL-8 in Lung Lavage Samples

All inflammatory mediators in lung lavage samples were analyzed using enzyme-linked immune-sorbent assay (ELISA) kits (R&D Systems, Wiesbaden-Norderstadt, Germany). All measurements were performed in triplicates. The assays were performed according to the manufacturer’s instructions. The data was stated as mean ± standard deviation (SD).

### 2.7. Analysis and Quantification of Ibuprofen in Porcine Plasma and Tissue Samples

Plasma and tissue samples were analyzed by high-performance liquid chromatography (HPLC) (Purospher Star, Merck KgaA, Darmstadt, Germany) and mass spectrometry (MS/MS; API4000, Applied Biosystems, Darmstadt, Germany). All samples were tested with the same equipment.

#### 2.7.1. Internal Standard

Naproxen was used as internal standard for the analysis of ibuprofen serum samples while ibuprofen-d3 was used as internal standard for analysis of ibuprofen tissue samples. Both naproxen and ibuprofen-d3 were dissolved in methanol in a concentration of 1 mg/mL. Twenty µL of this solution was then diluted with 9980 µL methanol/H_2_O (90 + 10, *v*/*v*) to a final concentration of 2 µg/mL.

#### 2.7.2. Calibration Standard

Ibuprofen was dissolved in methanol to a concentration of 1 mg/mL. Afterwards it was diluted with methanol/H_2_O to a final concentration of 100 µg/mL. From this stock solution, 10 calibration standard solutions were prepared: 20, 50 100, 200, 500, 1000, 2000, 5000, 10,000, and 20,000 ng/mL.

Ten µL aliquots of the 10 different calibration standard solutions were pipetted into 1.5 mL Eppendorf vials (Eppendorf AG, Hamburg, Germany) containing 100 µL porcine blank plasma as well as into 10 mL polypropylene tubes (NUNC Immuno tubes, Nalge, Rochester, NY, USA) containing 200 µg blank porcine lung homogenate. The final ibuprofen concentration of the calibration standards ranged from 2–2000 ng/mL in plasma and from 1–1000 ng/mL in lung homogenate.

ANALYST software (Applied Biosystem, version 1.4.2, Foster City, CA, USA) was used to calculate the calibration curves by linear regression analysis of the ibuprofen area ratio in relation to the internal standard. Ibuprofen calibration curves were linear over the entire calibration range (ibuprofen plasma: *y* = 0.00052 × *x* − 0.00202; *r*^2^ = 0.99213; ibuprofen lung homogenate: *y* = 0.00043 × *x* + 0.00102; *r*^2^ = 0.99448). Ibuprofen concentration of the test samples and the quality control samples were calculated on the base of the corresponding calibration curve.

#### 2.7.3. Quality Control Samples

Preparation of the ibuprofen quality control samples was performed analogous to the preparation of the calibration standard samples. Four quality control solutions were prepared from the stock solution with final ibuprofen concentrations of 150, 1500, 3000, and 15,000 ng/mL. The resulting ibuprofen concentrations in the final plasma samples ranged from 15 to 1500 ng/mL while the ibuprofen concentrations in the lung homogenate samples ranged from 7.5 to 750 ng/mL.

#### 2.7.4. Preparation of the Lung Homogenates

Porcine lung tissue samples were taken from the very dorsal areas of the right lower, medial, and upper lobe. For sample preparation, 3 mL of a phosphate buffer solution per 1 g of lung tissue was added to the samples. The mixture was then homogenized with the help of a Teflon potter (Sigma-Aldrich, Darmstadt, Germany), portioned and frozen at −20 °C till further analysis.

#### 2.7.5. Preparation of Ibuprofen Plasma Samples, Ibuprofen Lung Homogenate Samples, Calibration Standard, and Quality Control Samples

A 10 µL aliquot of the internal standard solution was pipetted into each test sample (porcine plasma, porcine lung homogenate, calibration standard, and quality control). The final concentration of naproxen in each ibuprofen serum samples was 200 ng/mL while the final concentration of ibuprofen-d3 in the ibuprofen lung homogenate samples was 500 ng/mL.

After spiking the test samples with the internal standard, 150 µL methanol was added. The samples were shaken and centrifuged for 15 min at 20,000× g. Aliquots of 100 µL were pipetted into HPLC vials, from which 50 µL was injected directly into the HPLC system.

For test drug analysis, some of the ibuprofen plasma samples had to be diluted with porcine blank plasma because plasma concentrations above the limit of quantification of the calibration curve were expected. The lower limit of quantification of the analytical method was approximately 10 ng/mL for undiluted plasma test samples and 4 ng/g for lung homogenate samples.

#### 2.7.6. HPLC and Mass Spectrometry Quality Control

Depending on the tested plasma and lung homogenate samples, ibuprofen quality control samples were analyzed together with each analytical batch to ensure consistent measurement quality. In each batch of test samples, quality control samples of all prepared concentration were tested right before and after the analysis of the test samples.

#### 2.7.7. Pharmacokinetic Analysis

The pharmacokinetic parameters following inhalation administration of ibuprofen were obtained by a noncompartmental analysis using WinNonlin Professional version 5.2 (Pharsight Corporation, Mountain View, CA, USA). The area under the curve (AUC_0–300min_) was calculated using the linear trapezoidal rule from time zero to the last measured postdose plasma concentration.

### 2.8. Statistical Analysis

Statistical analysis was performed using IBM SPSS Statistics 24 (2016, Armonk, NY, USA). The data of TNF- α, IL-6, and IL-8, ibuprofen lung tissue and serum concentrations showed a Gaussian distribution (*p* > 0.05 for both Kolmogorov–Smirnov test and Shapiro–Wilk test). ANOVA was performed in order to compare differences in the concentrations of the inflammatory mediators and the ibuprofen concentrations between the three groups. For post hoc analysis, we used Tukey’s test.

Lung mechanics and oxygenation index also showed a Gaussian distribution (*p* > 0.05 for both Kolmogorov–Smirnov test and Shapiro–Wilk test). To compare differences between the three groups, ANOVA was again performed with a Tukey’s test for post hoc analysis. To compare differences before and after lung injury within a group, a paired sample *t*-test was performed.

All graphs and figures were created using GraphPad Prism Version 6 (2012, GraphPad Software, San Diego, CA, USA).

## 3. Results

### 3.1. Acute Respiratory Distress Syndrome (ARDS) Model

Except for a significantly higher extravascular lung water (EVLW) in the sham group when compared to the F6H8 group before induction of ARDS (359 ± 20 mL vs. 304 ± 34 mL; *p* = 0.032), there were no statistically significant differences between the three groups regarding pre- or post-ARDS values of EVLW, peak pressure, transpulmonary pressure, static compliance, and PaO2/FiO2 ratio.

After induction of ARDS, EVLW increased from 344 ± 10 mL to 631 ± 19 mL, peak pressure from 17.35 ± 0.51 mbar to 35.9 ± 0.67 mbar and transpulmonary pressure from 3.41 ± 0.61 mbar to 15.97 ± 0.87 mbar. Static compliance decreased from 31.64 ± 0.92 mL/mbar to 17.94 ± 0.35 mL/mbar and PaO_2_/FiO_2_ ratio from 404.08 ± 18.81 mmHg to 52.55 ± 2.72 mmHg. EVLW, peak pressure, and transpulmonary pressure were significantly higher after induction of ARDS, while static compliance and PaO_2_/FiO_2_ ratio were significantly lower (*p* < 0.001 for all parameters).

At the end of the experiment, Ppeak decreased in the sham group (*p* = 0.02). In the F6H8 group, Ppeak was also significantly lower (*p* = 0.021). All other parameters did not differ significantly when compared to the ARDS onset.

A summary of all data is shown in Table 1 and Table 2.

### 3.2. Ibuprofen Lung Tissue Distribution

For the animals administered with ibuprofen (*n* = 8) the concentration of the compound was investigated at three different locations in the dependent part of the right lung. The ibuprofen tissue concentration in the lower lobe was 843.2 ± 236.3 ng/g, in the middle lobe 1373.5 ± 362.1 ng/g, and in the upper lobe 1036.3 ± 238.0 ng/g.

**Figure 1:**
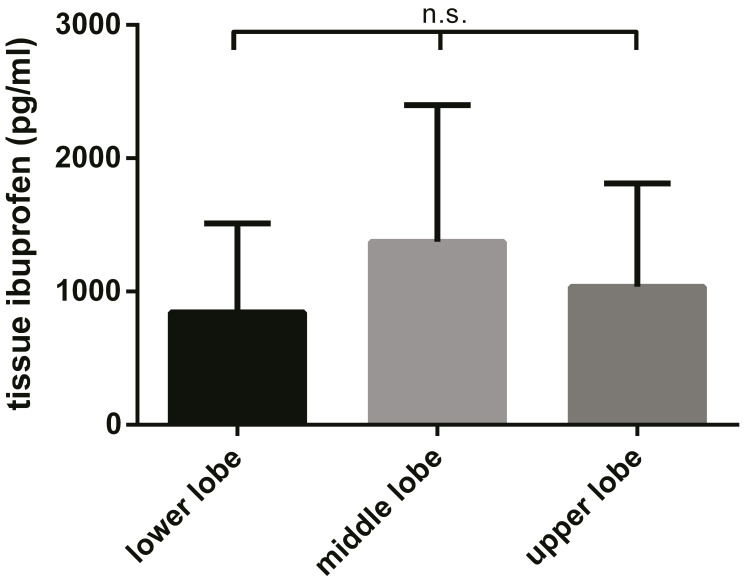
Ibuprofen concentration in the dependent parts of the three lobes of the right lung (mean ± SD for *n* = 24). The highest ibuprofen concentration was detected in the middle lobe, but no significant differences were observed between the three locations, indicating equal distributions throughout the lung tissue.

The highest ibuprofen concentration was detected in the middle lobe, but no significant differences were observed in the ibuprofen concentration between the three locations, indicating equal distributions of the compounds throughout the lung tissue, as shown in Figure 1.

### 3.3. Ibuprofen Plasma Concentration

**Figure 2:**
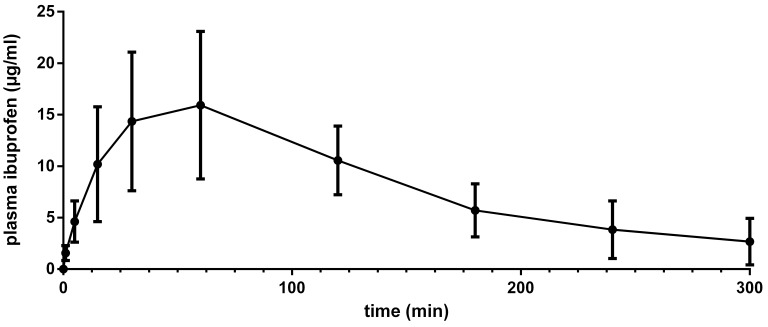
Plasma concentration profiles of ibuprofen as a function of time (mean ± SD for *n* = 8) for ibuprofen with a dose of 6 mg/kg. Ibuprofen was delivered with nebulized semifluorinated alkanes (SFAs) (F6H8). AUC_0-last_ was 2489 ± 916 min µg/mL, *C*_max_ 17.1 ± 7.4 µg/mL, and *t*_max_ 56 ± 30 min.

Plasma blood samples taken from the animals allowed for analysis of ibuprofen concentrations. The obtained plasma concentration profile is shown in Figure 2. This led to an AUC_0-last_ on 2489 ± 916 min µg/mL, a *C*_max_ on 17.1 ± 7.4 µg/mL, and a *t*_max_ on 56 ± 30 min (mean ± SD; *n* = 8).

### 3.4. Inflammatory Mediators in Bronchoalveolar Lavage Samples

#### 3.4.1. TNF-α Concentration in BAL Samples

**Figure 3:**
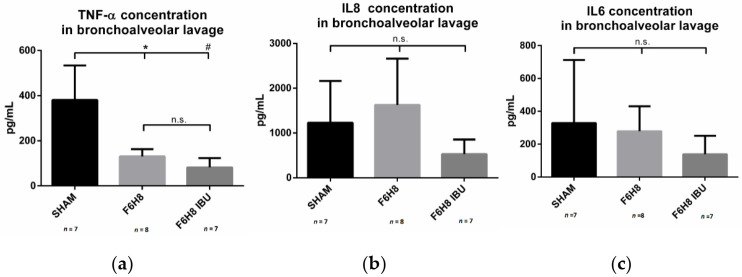
(**a**) TNF-α concentration in lung fluid after bronchoalveolar lavage: a significantly higher TNF-α concentration was observed in the sham group when compared to the F6H8 group (* *p* = 0.006) and F6H8-ibuprofen group (# *p* = 0.001). There was no significant difference in the TNF- α concentrations between the two additional groups. (**b**) IL-8 lung fluid concentration: no statistical difference was observed between the groups; however, a trend was observed toward a lower IL-8 level in the ibuprofen treatment animals. (**c**) IL-6 lung fluid concentration: no statistical difference was observed between the groups; but again, a trend was observed toward a lower IL-6 level in the ibuprofen-treated animals.

The mean concentration of TNF-α was 381 ± 77 pg/mL, 131 ± 13 pg/mL, and 82 ± 17 pg/mL in groups one to three, respectively, i.e., sham, F6H8 and F6H8-ibuprofen, see Figure 3a.

A significantly higher TNF-α concentration was observed in the sham group when compared to F6H8 (*p* = 0.006) and to F6H8-ibuprofen (*p* = 0.001). The difference in the TNF- α concentrations between the two F6H8 groups was not significant.

#### 3.4.2. IL-8 Concentration in BAL Samples

The mean concentration of IL-8 was 1229 ± 353 pg/mL, 1627 ± 462 pg/mL, and 528 ± 123 pg/mL, for the sham, F6H8, and F6H8-ibuprofen groups, respectively, see Figure 3b. For IL-8, there was no statistical difference between the groups; however, a trend toward a lower IL-8 level in the F6H8-ibuprofen group was observed.

#### 3.4.3. IL-6 Concentration in BAL Samples

The mean concentration of IL-6 was 329 ± 145 pg/mL, 646 ± 371 pg/mLs and 139 ± 46 pg/mL in groups one to three, respectively, i.e., sham, F6H8, and F6H8-ibuprofen, see Figure 3c. No statistical difference was observed between the groups; however, we noticed a trend toward lower IL-6 levels in the F6H8-ibuprofen group.

## 4. Discussion

The aim of the present animal study was to investigate whether an F6H8-aerosol is a suitable carrier for drug delivery not only to healthy lungs, as already demonstrated, but also to inhomogeneous ARDS lungs. The research on new drug delivery techniques to these lungs is of particular importance as they are characterized by normally aerated, overinflated, poorly aerated, and nonaerated regions, of which the latter are extremely unlikely to be reached by a conventional aerosol. For practical reasons, ibuprofen was used as paradigmatic drug. Ibuprofen plasma and lung tissue concentrations were measured, as well as the concentrations of inflammatory mediators in the lung fluid, such as TNF-α, IL-8, and IL-6. Lung mechanics were followed up and documented during the whole observation period to exclude negative side effects of the carrier compound.

The main findings are as follows: (1) F6H8 seems to be a suitable drug carrier in ARDS lungs. (2) F6H8-ibuprofen obviously reached poorly or even nonaerated lung regions as the lung tissue probes were taken from the most dependent parts of the lung. (3) The applied dose led to a provable ibuprofen plasma concentration, indicating that reasonable amounts of the drug passed the alveolo-capillary membrane and crossed the endothelial cells of the lung vasculature. (4) In the applied dosage of 1 mL/kg, F6H8 aerosol seemed to exert anti-inflammatory effects which were further pronounced when F6H8-ibuprofen was applied. (5) No negative side effects on lung mechanics were observed.

For the present study a well-established model for inducing ARDS in pigs was used [29]. ARDS criteria were met within an hour after induction of lung injury by surfactant depletion and injurious ventilation. The PaO2/FiO2 ratio decreased from physiological values to 52.55 ± 2.72 mmHg at a PEEP ≥ 5 mbar. The oxygenation index was below our cut-off value (defined as a PaO_2_/FiO_2_ < 100 mmHg) and met the Berlin definition indicating a severe ARDS [30]. The considerable increase in peak pressures and transpulmonary pressures indicated severe lung tissue alteration [31]. The marked increase of extravascular lung water measured with the transpulmonary thermodilution technique further confirmed severe lung edema as a result of lung damage [32].

### 4.1. Ibuprofen Delivery Using F6H8 as Drug Carrier

The pharmacokinetic data showed a systemic absorption of the pulmonary administered ibuprofen. The present work was intended as a proof-of-concept study to demonstrate drug transport across a pathologically altered alveolo-capillary membrane even in poorly or nonaerated lung areas. The study was not designed as a pharmaco-kinetic study which would compare plasma levels of inhaled ibuprofen with plasma levels of orally or intravenously administered ibuprofen. Reporting about bioavailability was outside of the scope of this study.

The area under the concentration-time curves (AUCs) reported in the present work were in line with those reported by Millecam and coworkers after administering 5 mg/kg ibuprofen intravenously and orally to pigs [33]. This suggests a complete absorption of the pulmonary administered ibuprofen, although these findings should be perceived cautiously since the type of animal, animal strains, bioanalysis, etc., may influence the results. Dembinski et al. dosed approximately 30 mg/kg ibuprofen to 33–34 kg pigs by a single intratracheal application with the compound dissolved in F6H8, i.e., a dose approximately six times as high than in the present study, and reported a systemic absorption of about 50%, suggesting an absorption that is substantial in accordance with the findings in the present study [24]. *t*_max_ of the present study was slightly higher compared to *t*_max_ after oral administration reported by Millencam et al. [33]. This indicates a faster pulmonary absorption, which is in line with the general learnings on drug delivery.

In summary, the findings of the present study indicate that a F6H8 aerosol might be a suitable carrier for pulmonary drug delivery in ARDS lungs. Nebulized ibuprofen seems not only to reach the alveolar epithelium and the interstitium but also the endothelium of the pulmonal vasculature. Most importantly, ibuprofen must have crossed the endothelium as it was systemically absorbed in a dosage comparable to oral administration, even leading earlier to the maximum plasma concentration. This was obvious proof of transportation across the cell layers of the alveolo-capillary membrane, suggesting that a high local concentration of any drug could be achieved when deliverable via a F6H8 aerosol.

A still open question is the extent to which the lowest TNF-α lung fluid concentration in the F6H8-ibuprofen group is due to a local ibuprofen effect on the cells when crossing the alveolo-capillary membrane or a systemic effect of the ibuprofen plasma levels [34].

### 4.2. Aerosol Distribution in the ARDS Lung

Many studies have examined aerosol deposition in dependency of lung physiology, but very few of them focused on aerosol distribution in ARDS lungs [35,36,37]. Inhomogeneity represents an immanent part of ARDS pathophysiology that hinders or even impedes conventional aerosol delivery as nonaerated lung regions are not be reached by an aerosol and poorly aerated lung regions only sparsely. In brief, only the “baby lung”, as the healthiest part of an ARDS lung, receives adequate aerosol treatment while wide parts of the pathologically altered regions and their epithelial surface remain inadequately reached and treated [17,38,39].

We found the highest ibuprofen tissue concentration in the dorsal part of the middle lobe, with concentrations in the range 843–1373 ng/g, while the average plasma concentration at the time of sampling was 2676 ng/mL. Determined after oral administration in humans, ibuprofen has an apparent volume of distribution (*V*d/*F*) of around 0.1 to 0.2 L/kg [40]. Assuming that the *V*d/*F* is similar in pigs, the observed lung concentration in the present study was relatively high after pulmonary administration using SFA as drug-delivery vehicle.

The ibuprofen tissue concentration in the dorsal part of the middle lobe was about 24.5 % higher than in the dorsal part of the upper lobe and 38.6 % higher than in the dorsal part of the lower lobe, which is consistent with ARDS pathophysiology. It is noteworthy that the ibuprofen concentration in the lower lobe was only 18.6% less than in the upper lobe, keeping in mind that in patients suffering from ARDS, the gas fraction of the lower lobe represents only 12 to 18% of the overall gas volume of the lung [41].

Tsagogiorgas and coworkers previously reported aerosol particle size and size distribution for both F6H8 and F6H8-ibuprofen [25,26]. Regarding F6H8 (*n* = 10), they found a mass median aerodynamic diameter (MMAD) of 2.98 ± 0.15 with 89% of droplets smaller than 5 µm and 43% of droplets smaller than 1 µm [26]. Regarding F6H8-ibuprofen (*n* = 12), MMAD was 2.29 ± 0.24 with 93% of droplets smaller than 5 µm and 45% of droplets smaller than 1 µm [25]. Both groups showed particle sizes and size distributions considered to be suitable for aerosol drug deposition to the small airways and alveoli [42]. Nevertheless, there was a significant difference between MMADs when comparing both groups (*p* < 0.001). We therefore cannot exclude that the significant differences in particle size between the two groups contributed to the observed differences in drug distribution within the lungs.

The unique physicochemical characteristics of SFAs such as a high or increased spreading coefficient, the reduction of surface tension, and a high specific weight might explain their potential to act as efficient and effective drug carriers in ARDS aerosol therapy, allowing one to reach lung areas not accessible for conventional gas-aerosol mixtures.

### 4.3. Influence of F6H8-Aerosol and F6H8-Ibuprofen on Inflammation

The inflammatory response in patients suffering from ARDS is triggered by the release of various cytokines and proinflammatory molecules [43]. Recent studies demonstrated that TNF-α as well as IL-6 and IL-8 were significantly increased in plasma and lung fluid and might be considered as biomarkers to predict mortality [44,45].

We found a significantly higher TNF-α lung fluid concentration in the control group when compared to the other two groups. TNF-α concentration was lowest in the F6H8-ibuprofen group, albeit there was no significant difference to the F6H8 group. Those findings might be explained by the anti-inflammatory effects of ibuprofen as well as of F6H8 itself [46,47].

Wheeler et al. hypothesized that the proinflammatory effects of TNF-α are at least partly caused by cyclooxygenase activation and demonstrated that ibuprofen in a dose of 14 mg/kg lowers pulmonal artery pressure and hypoxemia in TNF-α induced lung injury in sheep [48]. Carey et al. showed that a dose of 12.5 mg ibuprofen increased plasma TNF-α activity in a porcine sepsis model [46]. Consequently, we observed the lowest IL-8 and IL-6 lung fluid concentrations in the F6H8-ibuprofen group, even though there was no statistically significant difference to the other groups.

The significant lower TNF-α concentration in the F6H8 group could be explained by an anti-inflammatory effect of SFAs themselves. Van der Hardt et al. were able to demonstrate that PFC aerosol was able to suppress early pulmonary inflammatory response in a piglet ARDS model [47]. It is conceivable that the physicochemically closely related SFAs might exert the same suppressing effects on early inflammation. However, as an inhalation control group was not included, it cannot be distinguished whether the observed significant reduction in TNF-α was due to F6H8 itself or due to the inhalation of nebulized aerosol.

### 4.4. Influence of F6H8-Aerosol on Lung Mechanics

PFCs have been used for total as well as for partial liquid ventilation [49,50]. Studies have shown, amongst other things, an improvement in pulmonary function when used in ARDS lungs [23,50]. Kandler et al. first studied aerosolized PFCs in porcine ARDS models and were able to demonstrate a dose-dependent improvement in lung mechanics [51,52]. As SFAs are physicochemically closely related to PFCs, it seemed logical to evaluate their suitability for liquid ventilation [21].

Dembinski et al. were the first to use SFAs for partial liquid ventilation. Their work indicated an effect of SFAs on lung mechanics closely related to their dosage [24]. The assumption of dose-dependent effects of SFAs and PFCs on lung mechanics is also supported by the work of Kandler et al., which found an improvement in lung mechanics using considerably higher overall PFC dosages (5 mL kg^−1^ to 15 mL kg^−1^) [51].

The present study used a comparably low SFA dose (1 mL/kg). As previously expected, we were not able to observe an improvement in lung mechanics. However, and more importantly, the results exclude negative effects of such small SFA doses on lung mechanics supporting their suitability for drug delivery to inhomogeneous lungs.

### 4.5. Limitations

The study has two major limitations. Firstly, a drug control group to compare SFAs to other drug vehicles was not included. Due to its hydrophobicity, ibuprofen is poorly soluble in water [53]. To the best of our knowledge, there is no commercial ibuprofen inhalation solution available, which is supported by a recent review, that shows the limitations of aqueous ibuprofen solubility [54]. Therefore, we were unable to enroll a relevant nebulization control group. Secondly, since our study had no saline aerosol control group, it remains unclear, if the effect of the reduction in TFN-α occurred due to F6H8 itself or due to the inhalation of nebulized aerosol. Further, randomized controlled studies need to be conducted in order to investigate the potential anti-inflammatory effect of SFAs.

Another limitation of the present study is the absence of a lung-healthy control group. However, this work was designed as a proof-of-concept study, since we were unsure whether an F6H8 aerosol is suitable for drug delivery in a pathologically altered lung at all. For animal care reasons and basic ethical considerations (3R), the number of animals had to be limited to the three enrolled groups. Further studies will have to elucidate the differences in F6H8 and F6H8-ibuprofen aerosol deposition between healthy lungs and ARDS lungs, now that a benefit has been proven. In addition, it would have been certainly preferable to have taken and analyzed fluid and tissue samples not only from the most dorsal parts of the right lungs upper, middle, and lower lobes but also from the middle and most ventral parts of the ventro-dorsal axis. Lung histology samples might have opened more insights on the effects of F6H8 alone and the F6H8-ibuprofen aerosol. Nonetheless, the study finally achieved a unique proof of principle within the limitations presented.

## 5. Conclusions

The present study demonstrates that F6H8 aerosol seems to be a suitable carrier for pulmonary drug delivery to ARDS lungs using ibuprofen as paradigmatic “indicator” drug. Ibuprofen plasma concentration was high enough to exert anti-inflammatory effects, which led to the lowest TNF- α, IL-8, and IL-6 concentrations in the F6H8-ibuprofen group. Further studies will have to elucidate at which doses nebulized SFAs might have beneficial effects on respiratory mechanics and might even induce sustainable lung recruitment.

In a dosage of 1 mL/kg, F6H8 aerosol might have anti-inflammatory effects. Further studies are needed to clarify whether the observed effects were due to F6H8 itself or the inhalation of the nebulized aerosol. If F6H8 aerosol would show anti-inflammatory properties, it might help to suppress the early inflammatory response in patients developing an ARDS. “Classical” ARDS per se opens a wide window for the pulmonary application of drugs ranging from anti-inflammatory compounds to substances influencing the tone of the pulmonary vasculature or to drugs acting against the underlying cause, like antibiotics. The two latter approaches gain even more importance in face of the still-ongoing SARS-CoV-2 (COVID-19) pandemic, which is in part causing a pathophysiology that differs from the “classical” ARDS [55]. The specific pathophysiological changes of COVID-19-affected lungs offer a new attractive approach for local aerosol treatment, nebulizing compounds to reduce virus receptor binding, compounds to influence hypercoagulability in the pulmonary bed, or compounds to influence the vascular tone. This would allow a reduction in the large functional shunt of COVID-19 patients in the early phase as well as direct drug delivery to the endothelial cells of the pulmonary vascular bed [55,56,57,58].

In brief, this positive proof of principle study opens up a large range for research on compounds and treatment options using SFAs as carriers to improve the distribution of suitable drugs, especially in inhomogeneous lungs under various conditions.

## Figures and Tables

**Table 1:** Extravascular lung water (EVLW) and PaO_2_/FiO_2_ ratio pre- and post-lung injury to verify the successful induction of an acute respiratory distress syndrome (ARDS).

Group	EVLW (mL)		PaO2/FiO2 Ratio (mmHg)	
	Pre	Post	Pre	Post
Sham (*n* = 7)	358 ± 20 #	655 ± 45 *	430.7 ± 112.9	57.3 ± 14.3 *
F6H8 (*n* = 7)	304 ± 35	642 ± 91 *	446.8 ± 64.7	51.8 ± 8.9 *
F6H8-ibuprofen (*n* = 8)	339 ± 57	560 ± 106 *	391.4 ± 83.4	50.4 ± 9.5 *

* significant differences within group (pre vs. post). # significant differences between groups (Sham vs. F6H8 vs. F6H8-ibuprofen).

**Table 2:** Lung mechanics pre- and post-lung injury as well as at the end of the experiment.

Group	Peak Pressure (mbar)			Transpulmonary Pressure (mbar)			Compliance (mL/mbar)		
	Pre	Post	End	Pre	Post	End	Pre	Post	End
Sham (*n* = 7)	16.0 ± 2.9	35.3 ± 3.2 *	30.1 ± 3.2 #	3.4 ± 2.8	15.2 ± 3.5 *	13.0 ± 3.9	34.3 ± 5.8	18.2 ± 1.0 *	17.5 ± 5.9
F6H8 (*n* = 7)	17.0 ± 2.6	37.4 ± 3.6 *	35.43 ± 4.5 #	3.3 ± 3.0	15.1 ± 2.9 *	16.6 ± 7.8	32.0 ± 3.8	17.0 ± 1.4 *	16.7 ± 3.2
F6H8-ibuprofen (*n* = 8)	18.0 ± 1.7	37.6 ± 5.2 *	37.6 ± 3.6	2.5 ± 3.1	14.5 ± 6.5 *	16.1 ± 3.9	32.0 ± 4.1	17.5 ± 3.0 *	16.7 ± 2.5

* significant differences within group (pre vs. post). # significant differences within group (post vs. end).

## Data Availability

All data presented in this study is included in the submitted manuscript.

## Abbreviations

ARDS	acute respiratory distress syndrome
AUC	area under the concentration-time curve
BAL	bronchoalveolar lavage
*C*_max_	maximum concentration
HPLC	High-performance liquid chromatography
IL-6	interleukin 6
IL-8	interleukin 8
IV	intravenous
MMAD	mass median aerodynamic diameter
PEEP	positive end-expiratory pressure
PFC	perfluorocarbon
SFA	semifluorinated alkane
*t*_max_	time at which *C*_max_ was observed
TNF-α	tumor necrosis factor α
Vd/F	apparent volume of distribution after non-intravenous administration
ZEEP	zero end-expiratory pressure
